# Discovery of Donor-Derived Exosomal DNA as an Exploratory Biomarker of Kidney Graft Rejection: A Cross-Sectional Study

**DOI:** 10.3389/ti.2026.16061

**Published:** 2026-03-18

**Authors:** Elena Cuadrado-Payán, María José Ramírez-Bajo, Elisenda Banón-Maneus, Jordi Rovira, Natalia Hierro, Daniel Serrano-Jorcano, María Argudo, Enrique Montagud-Marrahi, Diana Rodríguez-Espinosa, Carolt Arana, Alicia Molina-Andújar, Angela González-Rojas, Nuria Esforzado, Vicens Torregrosa, Pedro Ventura-Aguiar, José Jesús Broseta, Eva González-Roca, Eduard Palou, Fritz Diekmann, David Cucchiari, Ignacio Revuelta

**Affiliations:** 1 Department of Nephrology and Kidney Transplantation, Hospital Clínic Barcelona, Barcelona, Spain; 2 Laboratori Experimental de Nefrologia I Trasplantament (LENIT), Fundació de Recerca Clínic Barcelona-Institut d’Investigacions Biomèdiques August Pi I Sunyer (FRCB-IDIBAPS), Barcelona, Spain; 4 Red de Investigación Renal (REDINREN) and RICORS2040, Instituto de Salud Carlos III, Madrid, Spain; 3 Department of Medicine, Faculty of Medicine and Health Sciences, University of Barcelona, Barcelona, Spain; 5 CORE Molecular Biology Laboratory, Biomedical Diagnostic Center (CBD), Hospital Clínic Barcelona, Barcelona, Spain; 6 Department of Immunology, Hospital Clínic Barcelona, Barcelona, Spain

**Keywords:** biomarker, exosomal DNA, exosomes, extracellular vesicles, kidney transplant

## Abstract

Circulating donor DNA has emerged as a valuable tool for clinical decision-making in kidney transplantation. While most studies focus on cell-free DNA, the role of donor DNA associated with extracellular vesicles (EVs) remains unexplored. To address this, we analyzed donor-derived exosomal DNA (dd-exoDNA) in 100 kidney transplant recipients (KTR) undergoing surveillance or indicated biopsies. Serum exosomes were isolated using precipitation-based technology, and dd-exoDNA was analyzed via digital PCR targeting donor/recipient HLA-DRB1 mismatches. Dd-exoDNA levels were higher in rejection versus non-rejection (2.66 [0.56–7.10] ×10^−3^ vs. 0.69 [0.28–1.71] ×10^−3^, *p* = 0.004) and were associated with Banff score items: glomerulitis ≥1 (*p* = 0.037), peritubular capillaritis ≥1 (*p* = 0.040), and tubulitis ≥2 (*p* = 0.043). In multivariate analysis, dd-exoDNA remained independently associated with rejection, although with wide confidence intervals (OR [95%CI] 3.68 [1.32–10.26], P = 0.013). Exploratory threshold analyses suggested moderate discriminative performance. These findings indicate that donor DNA associated with circulating EVs may offer complementary information to existing biomarkers, warranting validation in external cohorts and comparison with established assays.

## Graphical Abstract

**Figure:**
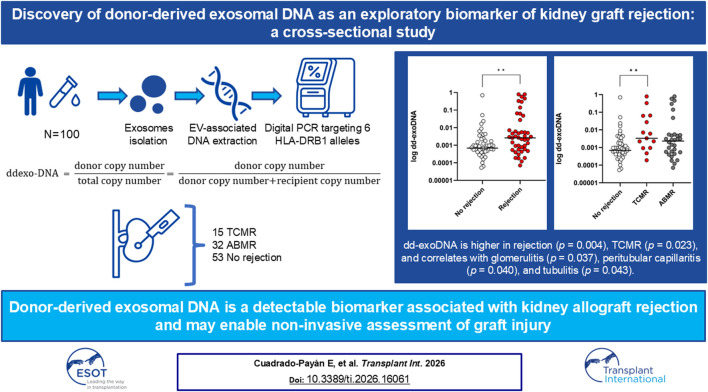

## Introduction

Early identification of allograft rejection is essential for timely treatment and improved graft outcomes. Although biopsy remains the diagnostic gold standard, its invasive nature limits both routine and repeated use [1]. Consequently, recent efforts have focused on non-invasive biomarkers to enable earlier detection of rejection [1–3].

While most studies have focused on circulating cell-free DNA (cfDNA) as a non-invasive biomarker, its performance is limited by variable specificity. Although emerging approaches allow inference of tissue of origin, sensitivity remains suboptimal, particularly for T cell–mediated rejection (TCMR) [4].

In this context, extracellular vesicles (EVs), particularly exosomes, have emerged as valuable sources of biologically informative circulating signals [5, 6]. Produced in the endosomal compartment of both resting and activated cells, exosomes play a crucial role in intercellular communication [6, 7]. They carry a diverse molecular cargo, including nucleic acids, proteins, and lipids, that reflects the molecular landscape of the parental cells [8]. In kidney transplantation, exosomes mediate innate and adaptive immune responses [9, 10], facilitating antigen presentation via the semi-direct pathway and transmitting allogeneic or tolerogenic signals, highlighting their involvement in immune processes relevant to graft rejection [9–14]. Building on this, exosomes have been shown to participate in tissue repair [15], regulate fibrosis [16], modulate renal injury [17], and reflect residual humoral immunity after desensitization [18]. However, exosomes should be interpreted as dynamic mediators of intercellular signaling rather than direct indicators of cell death or tissue injury.

Notably, exosomes have been shown to carry circulating DNA protected from degradation by their phospholipid bilayer, although EV-associated DNA may also localize on the vesicle surface [19–23]. While exosome DNA has shown promise as a biomarker in certain types of tumors [24–26], infections [19, 27], pregnancy [28], and prenatal diagnosis [29], its biological function and diagnostic value in solid organ transplantation remain largely unexplored, particularly regarding its potential to monitor kidney graft health.

Given these gaps, our study was designed as an exploratory, proof-of-concept evaluation to investigate the existence, detectability, and preliminary diagnostic relevance of donor-derived exosomal DNA (dd-exoDNA) by identifying HLA-DRB1 mismatches within circulating exosomes as an unequivocal signature of the donor’s origin [30] and evaluating whether this signal correlates with biopsy-proven rejection, the clinical gold standard.

## Materials and Methods

### Patient Recruitment and Sample Collection

This single-center, cross-sectional study conducted at Hospital Clínic Barcelona analyzed serum and histological samples from KTRs who underwent graft biopsies between 2018 and 2023. Inclusion criteria required age >18 years, a pathological diagnosis other than borderline rejection, and HLA-DRB1 alleles with available commercial probes. Exclusion criteria included pregnancy, multi-organ or dual kidney transplants, donor–recipient pairs identical for all four HLA-DRB1 alleles that could not be distinguished using this panel, and borderline rejection due to heterogeneity and limited diagnostic reproducibility. In patients with previous grafts, prior HLA-DR was not shared with the current donor.

Biopsies were performed for routine surveillance at 3 and 12 months post-transplantation or for clinical indications suchs as declining renal function, increasing proteinuria, or newly detected donor-specific antibodies (DSAs). Both surveillance and for-cause biopsies were included to evaluate whether dd-exoDNA correlates with histologic rejection, regardless of clinical presentation. Analyses were stratified by biopsy indication to account for potential differences in biomarker performance.

Serum samples were collected immediately before biopsy in serum separator tubes with gel separator (BD Vacutainer SST), aliquoted, and stored at −80 °C until processing.

Comprehensive demographic, clinical, laboratory, and immunological data were collected from electronic health records. The Pathology Department at Hospital Clínic de Barcelona carried out histological assessments of all biopsy samples, strictly adhering to the 2019 Banff classification criteria [31].

The study adhered to the Declaration of Helsinki. Pre-analytical procedures for sample collection, handling, and storage adhered to the guidelines established by the International Society for Extracellular Vesicles [32]. The local Ethical Committee approved the study (registry code HCB/2022/0044). All transplant procedures were performed in accordance with the Declaration of Istanbul.

### Exosome Isolation, Quantification, and Characterization

Exosomes were isolated using the precipitation method with the ExoGAG kit (Nasasbiotech, Spain), which interacts with surface glycosaminoglycans [33]. Serum samples were centrifuged at 2000g for 10 min. 2 mL of supernatant were mixed with ExoGAG reagent at a ratio of 1:2, incubated at 4 °C for 5 min, and centrifuged at 23,500 g for 30 min. EV pellet was resuspended in 200 μL of 0.1 μm-filtered phosphate-buffered saline (PBS).

Particle concentration and size distribution were determined by nanoparticle tracking analysis (NTA) using a NanoSight LM10 instrument (Malvern, UK) equipped with a 488 nm Blue laser and a SCMOS camera. For each sample, five 50-second videos were captured. Data were analyzed with NTA software v3.4. Exosomes’ morphology was assessed by transmission electron microscopy (TEM), as previously described by our group [34, 35].

Phenotypic characterization was performed using the MACSPlex Human Exosome Kit (Miltenyi Biotec), following the manufacturer’s recommendations. A total of 1 × 10^9^ EVs were incubated with 15 µL of antibody-coated beads overnight, followed by APC-labeled antibodies against CD9, CD63, and CD81 for 1 h. Data were acquired with a BD LSRFortessa SORP cytometer and analyzed using FACS DIVA software (BD Biosciences). Median fluorescence intensity (MFI) for each marker was normalized to average tetraspanin levels per sample.

### Exo-DNA Isolation and Pre-Amplification

ExoDNA was extracted using QIAamp DNA Mini Kits (Qiagen), following the manufacturer’s instructions, including protease treatment to reduce non-vesicular protein–DNA complexes potentially co-isolated during ExoGAG precipitation. No DNase I treatment of intact EVs was performed prior to lysis; therefore, the assay captures total EV-associated DNA, including both intravesicular DNA and DNA associated with the vesicle surface. Concentration and DNA integrity were assessed using Qubit single-strand molecular probes (Invitrogen) and an Agilent 4200 TapeStation System (Agilent), respectively, at the Functional Genomics Platform of the August Pi Sunyer Biomedical Research Institute (IDIBAPS). Quality thresholds were verified, with a main peak at ∼160–180 bp. DNA quantity was normalized to EV yield and stored at −80 °C.

### HLA-Specific Probes

HLA alleles in exoDNA were detected and quantified via dPCR using a panel of six TaqMan probes targeting specific HLA-DRB1 alleles (Bio-Rad): HLA-DRB1*01, HLA-DRB1*07, HLA-DRB1*08, HLA-DRB1*11, HLA-DRB1*13, and HLA-DRB1*15, based on Zout et al [36]. Each probe had HEX and FAM versions, enabling simultaneous detection of donor and recipient alleles. Of note, the HLA-DRB1*15 assay was also specific for the HLA-DRB1*16 allele. As previously described, 2 ng of exoDNA were pre-amplified using SSoAdvancedPreAmp Supermix (Bio-Rad) [36, 37].

### QuantStudio Absolute Q Digital PCR Assay

Digital PCR assays were conducted using the QuantStudio Absolute Q platform (Thermo Fisher Scientific), with microfluidic array plates for sample partitioning, following the protocol reported by Zou et al [36] and the HLA Expert Design datasheet. Each 9 µL reaction included 1X dPCR Master Mix, allele-specific probes, and exoDNA. PCR cycling conditions: 95 °C for 3 min, 40 cycles of 94 °C for 10s and 55 °C for 30s, followed by 72 °C for 5 min. Data were analyzed using the Absolute Q Digital PCR Software.

### Fraction Calculation

The percentage of dd-exoDNA was calculated according to the following formula, as previously described for dd-cfDNA [36]:

$$\text{dd}‐\text{exoDNA}\left(\text{fraction}\right)=\frac{\text{donor}\;\text{copy}\;\text{number}}{\text{total}\;\text{copy}\;\text{number}}=\frac{\text{donor}\;\text{copy}\;\text{number}}{\text{donor}\;\text{copy}\;\text{number}+\text{recipient}\;\text{copy}\;\text{number}}$$

The number of copies for alleles present with two copies per genome was adjusted to a single copy per diploid genome. For alleles shared by the donor and recipient, calculations were adjusted assuming equal allele contributions per genome.

### Statistical Analysis

Due to the exploratory nature of the study and the absence of prior dd-exoDNA data, no formal sample size calculation was performed. The cohort included 100 patients with well-defined histological diagnoses, comparable to those in early-phase biomarker studies [38]. The Kolmogorov-Smirnov test was used to assess normality. Normally distributed variables are presented as mean ± standard deviation (SD), and non-normally distributed variables as median and interquartile range (IQR). Qualitative variables are expressed as absolute and relative frequencies.

Between-group comparisons used Student’s t-test, Mann-Whitney U, or Kruskal-Wallis tests, and χ^2^or Fisher’s exact test as appropriate. When the Kruskal–Wallis test was applied, post-hoc pairwise comparisons were conducted using Dunn’s test with Bonferroni correction for multiple comparisons. ROC curves were used to evaluate diagnostic performance (AUC, 95% CI), with optimal thresholds determined by Youden’s Index. Associations were assessed with Spearman correlation.

For the MACSPlex analysis of 37 surface markers, unadjusted p values were first calculated using the Mann–Whitney U test. To control the false discovery rate, p values were adjusted using the Benjamini–Hochberg procedure; both unadjusted and adjusted p values are reported.

The clinical utility of dd-exoDNA was evaluated using Decision Curve Analysis (DCA) with logistic regression models including DSA and eGFR, with or without dd-exoDNA. Covariates for multivariable logistic regression were selected *a priori* based on clinical relevance. Sensitivity analyses confirmed robustness. No correction for multiple comparisons was applied for Banff lesion analyses due to the exploratory design; therefore, these findings should be interpreted as hypothesis-generating.

Statistical analyses were performed using IBM SPSS Statistics v31.0 (IBM Corp., Armonk, NY), except for DCA, which was conducted using the rmda package in R (v4.4.1). Graphs were generated with GraphPad v9.5.1 (GraphPad Software, La Jolla, CA). Two-tailed *p* < 0.05 was considered statistically significant.

## Results

### Baseline Characteristics of the Population

This study included serum and allograft biopsies from 100 KTR, comprising 34 surveillance and 66 clinically indicated biopsies performed at a median of 4 [1–13.5] months post-transplant. The baseline characteristics of the included patients are summarized in Table 1.

**TABLE 1:** Main baseline characteristics of the included patients.

Variable	N = 100
Recipient age at transplantation (years)	58.7 ± 14.6
Sex (male)	64 (64)
Recipient race	
Caucasian	85 (85)
Hispanic	10 (10)
Black	3 (3)
Asian	2 (2)
Etiology of CKD	
Unknown or uncertain	26 (26)
Glomerular disease	21 (21)
Urological	14 (14)
ADPKD	12 (12)
Diabetic nephropathy	10 (10)
Congenital nephropathy	8 (8)
Nephroangiosclerosis	6 (6)
Other	4 (4)
Interstitial nephropathy	3 (3)
Previous transplant	
0	80 (80)
1	16 (16)
2	3 (3)
3	1 (1)
cPRA	
0%	44 (44)
1%–49%	18 (18)
50%–89%	15 (15)
90%–98%	10 (10)
99%–100%	13 (13)
Donor type	
Living	24 (24)
Brain death	33 (33)
Type II circulatory death	9 (9)
Type III circulatory death	34 (34)
Induction immunosuppression	
Thymoglobulin	44 (44)
Basiliximab	56 (56)
Maintenance immunosuppression	
Prednisone + Tacrolimus + MMF	39 (39)
Prednisone + Tacrolimus + mTORi	61 (61)
Histological diagnosis	
Nonspecific changes	28 (28)
IFTA	16 (16)
Chronic vascular changes	7 (7)
Diabetes-related complications	2 (2)
ABMR	32 (32)
TCMR	15 (15)
DSAs at biopsy	16 (16)

Data are expressed as mean ± standard deviation or absolute number and percentage (ADPKD, autosomal dominant polycystic kidney disease; CKD, chronic kidney disease; cPRA, panel reactive antibodies; DSA, donor-specific antibodies; MMF, Mycophenolate mofetil; mTORi, mTOR inhibitors; IFTA, interstitial fibrosis with tubular atrophy; ABMR, antibody-mediated rejection; TCMR, T cell-mediated rejection).

The mean age of this cohort at the time of transplant was 58.71 ± 14.6 years, with predominantly male sex (64.0%) and recipients of a first transplant in 80.0% of cases. Thirty-eight patients had positive panel reactive antibodies (cPRA) ≥50% at the time of biopsy.

Histopathological analysis revealed rejection in 47 biopsies. Among these, 15 were TCMR, and 32 were classified as antibody-mediated rejection (ABMR). No mixed rejection cases were identified. Of the ABMR cases, 11 met Banff criteria and had detectable DSAs. The remaining 21 cases were classified as ABMR based on the presence of microvascular inflammation and/or positive C4d staining. TCMR cases were further classified into 8 acute and 7 chronic-active. Regarding ABMR, 22 cases were active, and 10 were chronic-active.

Among the 53 biopsies without evidence of rejection, the most frequent findings were non-specific changes (28 cases) and interstitial fibrosis with tubular atrophy (IFTA, 16 cases). Other diagnoses included chronic vascular changes (7 cases) and diabetes-related complications (2 cases).

### Quantitative and Qualitative Evaluation of Circulating Serum Exosomes

**FIGURE 1:**
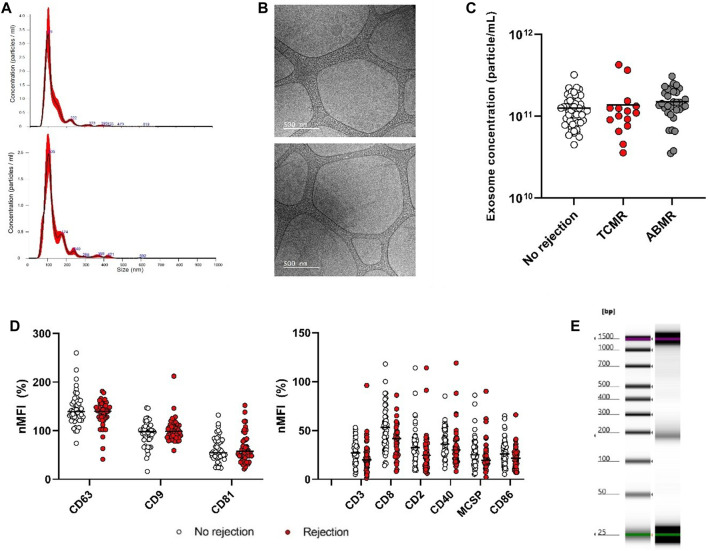
Quantification and characterization of serum exosomes (TCMR; T cell-mediated rejection, ABMR; antibody-mediated rejection, nMFI; normalized median fluorescence intensity). One-way Kruskal-Wallis test followed by multiple pairwise comparisons was used. **(A)** Representative size distributions of exosomes in one sample from the rejection (upper panels) and the non-rejection group (lower panels) with Nanoparticle Tracking Analysis. **(B)** Transmission electron microscopy visualization of the grid with an irregular distribution of hole sizes and shapes containing exosomes of various sizes (scale bars 500 nm) from one sample from the rejection (up) and the non-rejection group (down). **(C)** Quantification of serum exosomes depending on biopsy results, showing a non-significant higher concentration in ABMR cases (p = 0.065). **(D)** MFI signal of tetraspanins (left) and common immune cell-derived surface exosomes’ antigens (right) detected using the MACSPlex kit according to rejection or not (only 6 out of 37 surface antigens that displayed a nominal (unadjusted)difference between the two groups are shown). **(E)** Electrophoresis of exo-DNA sample, with electronic ladder showing specific sizing standards (left) and exo-DNA showing an average DNA fragment length ranging between 160 and 200 bp (right).

Serum exosomes were quantified by NTA, and TEM revealed their typical round shaped membrane particles (Figures 1A,B).

Exosomes concentration was 1.26 × 10^11^ [0.883 × 10^11^-1.67 × 10^11^] particles/mL. When stratified by biopsy results, exosome concentrations were numerically higher in cases with rejection compared to those without (1.30 × 10^11^ particles/mL (9.8 × 10^10^–1.84 × 10^11^ particles/mL) vs. 1.16 × 10^11^ particles/mL (8.3 × 10^10^–1.51 × 10^11^ particles/mL), although this difference did not reach statistical significance (*p* = 0.19). Further differentiation between cellular and humoral rejection revealed a similar trend toward higher exosome concentrations in ABMR compared to TCMR (1.4 × 10^11^ [1.16 × 10^11^–1.98 ×10^11^] vs. 1.1 × 10^11^ [8.3 × 10^10^–1.3 × 10^11^] particles/mL; *p* = 0.065) (Figure 1C).

Further exosome characterization was performed using the MACSPlex kit, which analyzes 37 common surface and immunological exosome biomarkers. Several markers showed unadjusted differences between rejection and non-rejection groups, with lower median expression levels observed in the rejection group for CD3, CD8, CD2, CD40, MCSP, and CD86 (Figure 1D). When stratified by rejection type, CD40 serum exosome antigen was also significantly lower in patients with ABMR than in those without rejection. However, after adjustment for multiple comparisons, no marker remained statistically significant (Supplementary Table S1).

### Quantification of exoDNA in Stable and Rejection Patients

The average DNA fragment length of exo-DNA ranged between 160 and 200 base pairs (bp) (Figure 1E), while the median exoDNA concentration was 0.917 [0.48–1.50] ng/µL. No differences were observed in terms of exoDNA concentrations between patients with and without rejection (1.01 [0.49–1.56] ng/μL for control versus 0.83 [0.37–1.48] ng/μL for rejection (*p* = 0.62). Additionally, exoDNA concentrations were higher in TCMR than in ABMR (1.32 [0.58–2.33] vs. 0.75 [0.36–1.40] ng/μL), although this difference was not statistically significant (*p* = 0.32).

### Detection of Donor-Derived ExoDNA (Dd-ExoDNA) and Association With Kidney Graft Rejection

**FIGURE 2:**
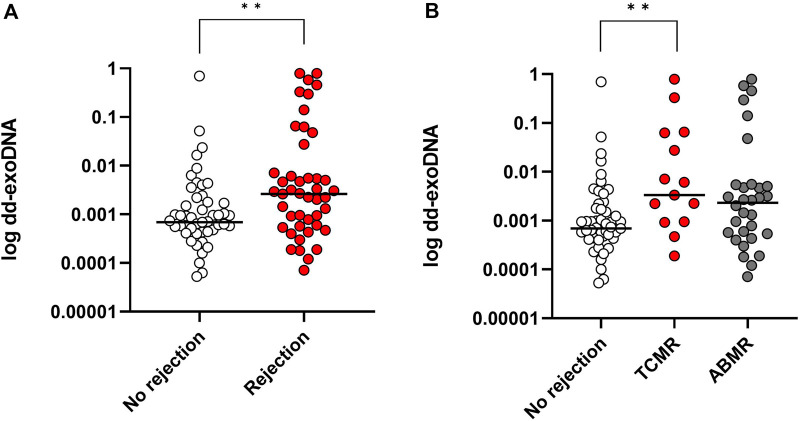
Dd-exoDNA fraction levels according to biopsy results (TCMR; T cell-mediated rejection, ABMR; antibody-mediated rejection). **(A)** Higher dd-exoDNA fraction levels were observed in rejection cases (p = 0.004) compared to no-rejection. **(B)** A significant difference between no-rejection and TCMR group is seen (p = 0.022). One-way Kruskal-Wallis test followed by multiple pairwise comparisons was used.

Dd-exoDNA was consistently detected in all patients 0.92 [0.47–1.51] × 10^−3^). No differences were observed between living donors and deceased donors (1.02 [0.58–3.6] ×10^−3^ vs. 0.92 [0.40–4.56] ×10^−3^, *p* = 0.35), nor among the different types of deceased donors. In patients with any-type rejection, dd-exoDNA was significantly higher (2.66 [0.56–7.10] × 10^−3^) compared to patients without rejection (0.69 [0.28–1.71] × 10^−3^), with a *p* = 0.004 (Figure 2).

Dd-exoDNA levels showed substantial dispersion within the TCMR group, particularly in chronic-active TCMR, with a right-skewed distribution characterized by a subset of cases exhibiting markedly elevated values. In line with this heterogeneity, the global Kruskal–Wallis test did not reach statistical significance (*p* = 0.09); however, post-hoc pairwise comparisons identified a significant difference between the no-rejection and TCMR groups (*p* = 0.022), while no significant differences were observed for the remaining comparisons (Table 2).

**TABLE 2:** Dd-exoDNA levels across different rejection categories.

Rejection category	Dd-exoDNA fraction	p value
No rejection (n = 53)	0.69 [0.28–1.71] × 10^−3^	Ref
Rejection (n = 47)	2.66 [0.56–7.10] × 10^−3^	**0.004**
TCMR (n = 15)	3.32 [0.93–45.03] × 10^−3^	**0.022**
Active (n = 8)	2.78 [0.71–34.38] × 10^−3^	0.95
Chronic-active (n = 7)	7.09 [1.57–178.74] × 10^−3^	0.17
ABMR (n = 32)	2.01 [0.49–4.89] × 10^−3^	0.13
Active (n = 22)	1.20 [0.40–4.74] × 10^−3^	1
Chronic-active (n = 10)	3.17 [1.32–5.55] × 10^−3^	0.25

(TCMR, T cell-mediated rejection; ABMR, antibody-mediated rejection; Ref: reference). Data are expressed as median [interquartile range]. One-way Kruskal-Wallis test followed by multiple pairwise comparisons was used. Significantly higher levels of dd-exoDNA were observed among patients with rejection, particularly in those with TCMR. Median dd-exoDNA levels tended to be higher in chronic-active forms, although pairwise comparisons did not reach statistical significance. Bold values indicate statistically significant results (p < 0.05).

To evaluate whether extreme observations drove these findings, a sensitivity analysis excluding outliers identified using an IQR–based rule applied to log-transformed dd-exoDNA values was performed. After exclusion, the difference between the TCMR and non-rejection groups was attenuated, indicating that a limited subset of TCMR cases accounted for the elevated dd-exoDNA levels.

Among patients with ABMR, no significant differences in dd-exoDNA levels were observed between DSA-negative and DSA-positive cases (2.63 [0.40–5.43] × 10^−3^ vs. 2.02 [0.77–4.06] × 10^−3^, respectively; *p* = 0.755).

### Donor-Derived exoDNA (Dd-ExoDNA) Fraction and Banff Classification Scores

**FIGURE 3:**
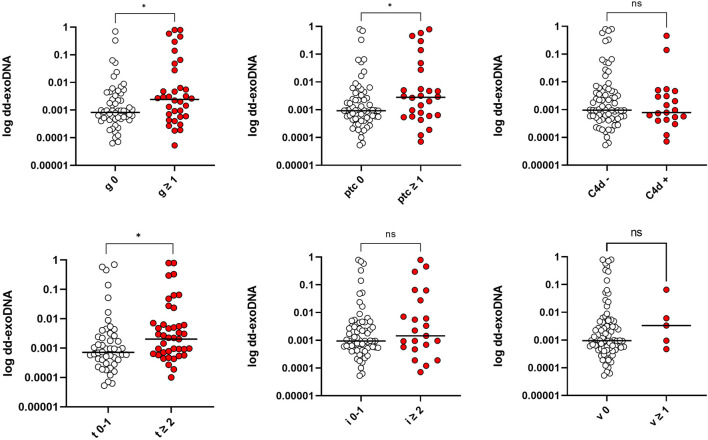
Relationship between dd-exoDNA fraction and individual Banff classification scores (g; glomerulitis, ptc; peritubular capillaritis, t; tubulitis, i; interstitial inflammation, v; vasculitis). Significant differences in dd-exoDNA were observed for glomerulitis (p = 0.037), peritubular capillaritis (p = 0.040), and tubulitis (p = 0.043), but no significant differences were found for complement fraction C4d deposition (p = 0.807), interstitial inflammation (p = 0.45), and vasculitis (p = 0.253). Mann-Whitney U test was used.

The relationship between dd-exoDNA fraction and individual Banff classification scores was also assessed. The median dd-exoDNA was 0.81 [0.40–2.42] × 10^−3^ in the absence of glomerulitis (g), while it was significantly higher (2.43 [0.50–16.87] × 10^3^) in those with g ≥ 1 (*p* = 0.037). Similarly, for peritubular capillaritis (ptc), the median dd-exoDNA was 0.92 [0.29–2.63] × 10^−3^ in samples with no inflammatory activity, increasing to 2.82% [0.58–16.47] × 10^−3^ in patients with ptc ≥1 (*p* = 0.040). For tubulitis (t), a significant difference was also observed, with values reaching 2.93 [1.19–17.23] × 10^−3^ in cases with moderate to severe (t ≥ 2), compared to 0.89 [0.40–4.36] × 10^−3^ in patients with mild or absent involvement (*p* = 0.043). In contrast, no significant differences were found regarding complement fraction C4d deposition (*p* = 0.807), interstitial inflammation (i) (*p* = 0.45), and vasculitis (v) (*p* = 0.253) (Figure 3).

### Diagnostic Performance of Donor-Derived ExoDNA (Dd-exoDNA)

**FIGURE 4:**
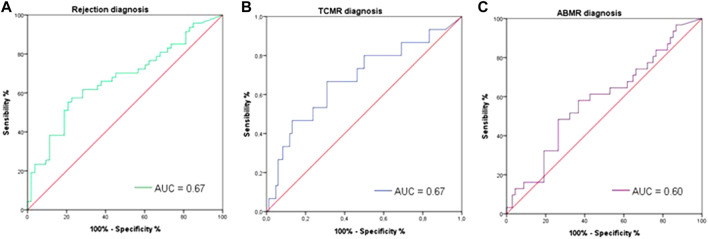
ROC curves for rejection diagnosis of dd-exoDNA fraction. (TCMR; T cell-mediated rejection, ABMR; antibody-mediated rejection). **(A)** An area under the curve of 0.67 (95% CI: 0.56–0.79) for distinguishing rejection from non-rejection is seen (green line). **(B)** For diagnosis of TCMR, dd-exoDNA shows an area under the curve of 0.67 (95% CI: 0.51–0.83) (blue line). **(C)** For ABMR, the area under the curve is 0.60 (95% CI: 0.48–0.73) (purple line).

To assess the diagnostic performance of dd-exoDNA in distinguishing rejection from non-rejection, the area under the ROC curve (AUC) was calculated. The AUC for discriminating rejection from non-rejection was 0.67 (95% CI: 0.56–0.79). For differentiating TCMR or ABMR specifically from non-rejection, dd-exoDNA demonstrated an AUC of 0.67 (95% CI: 0.51–0.83) and 0.60 (95% CI: 0.48–0.73), respectively (Figure 4).

Compared with DSAs’ diagnostic ability at the time of biopsy, dd-exoDNA showed numerically higher AUC, although the confidence intervals overlapped, as DSAs yielded an AUC of 0.59 (95% CI: 0.47–0.70). Similarly, the estimated glomerular filtration rate (eGFR) showed poor diagnostic accuracy, with an AUC of only 0.37 (95% CI: 0.23–0.51) (Supplementary Figure S1).

Notably, dd-exoDNA performance varied depending on biopsy indication. In clinically indicated biopsies, its discriminative ability improved significantly (AUC 0.70 [95% CI: 0.57–0.83]), whereas for surveillance biopsies, the AUC was lower (0.52 [95% CI: 0.30–0.74]) (Supplementary Figure S2). It should be noted that among the 34 surveillance biopsies, only 5 were rejection cases, compared with 42 among the 66 clinically indicated biopsies.

We aimed to determine a clinically significant threshold for identifying samples potentially indicative of kidney graft rejection. Using a threshold of 1.9 × 10^−3^, dd-exoDNA showed a specificity of 77.4% and a sensitivity of 57.4% for distinguishing rejection from non-rejection. Among the 100 dd-exoDNA determinations, 39 were above the 1.9 × 10^−3^ threshold. Of these, 27 corresponded to patients with biopsy-proven rejection, while the remaining 12 were from patients without rejection. The positive predictive value (PPV) for identifying rejection was 69.2%, while the negative predictive value (NPV) was 67.2%. Among patients without histological rejection, those with exoDNA values above the 90th percentile showed heterogeneous biopsy findings. Two patients presented chronic graft lesions, including moderate chronic vascular changes and moderate interstitial fibrosis with tubular atrophy. In contrast, the remaining three showed no histological evidence of rejection or other relevant abnormalities.

**FIGURE 5:**
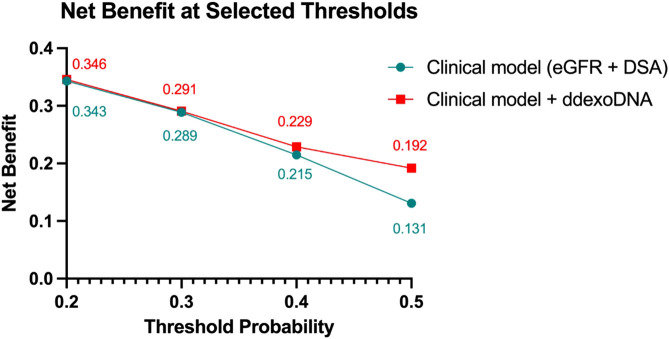
Decision Curve Analysis of Predictive Models. (eGFR: estimated glomerular filtration rate, DSA: donor-specific antibodies). Decision Curve Analysis comparing the net benefit of the clinical model (eGFR + DSA) versus the combined model including dd-exoDNA. The model with dd-exoDNA showed higher net benefit across clinically plausible thresholds (e.g., 0.20–0.30). Higher thresholds are shown for completeness.

To further assess whether dd-exoDNA fraction was independently associated with rejection, we conducted a multivariable logistic regression analysis adjusting for relevant clinical and immunological variables. In the adjusted model, dd-exoDNA remained independently associated with rejection, with an odds ratio (OR) of 3.68 (95%CI 1.32–10.26, *p* = 0.013). Biopsies performed for clinical indication were also significantly associated with rejection (OR 7.43, 95% CI 2.21–25.02, *p* = 0.001). Maintenance immunosuppression with mTOR inhibitors, although associated with a lower risk of rejection in the univariable analysis (OR 0.32, *p* = 0.007), did not retain statistical significance after adjustment (OR 0.4, *p* = 0.101). Likewise, eGFR at biopsy and DSAs at biopsy did not remain statistically significant after adjustment (Table 3). Decision curve analysis showed that the model including dd-exoDNA provided a higher net benefit than the baseline model (DSA + eGFR) across a range of probability thresholds (Figure 5).

**TABLE 3:** Univariable and multivariable logistic regression analysis of factors associated with rejection (cPRA, panel reactive antibodies; DSA, donor-specific antibodies; mTORi, mTOR inhibitors; MMF, Mycophenolate mofetil; eGFR, estimated glomerular filtration rate; OR, odds ratio).

Variables	Univariable		Multivariable	
	OR [95% CI]	p value	OR [95% CI]	p value
Sex (male)	0.98 [0.43–2.23]	0.97	​	​
Age	1.00 [0.98–1.03]	0.73	​	​
Previous transplants	0.70 [0.26–1.89]	0.48	​	​
cPRA	1.00 [0.99–1.01]	0.67	​	​
Donor type (deceased vs. living)	0.68 [0.27–1.72]	0.42	​	​
Induction immunosuppression (thymoglobulin vs. basiliximab)	1.55 [0.69–3.44]	0.28	​	​
Maintenance immunosuppression (mTORi vs. MMF)	0.32 [0.14–0.73]	**0.007**	0.4 [0.15–1.18]	0.101
Biopsy indication (clinical vs. surveillance)	10.15 [3.47–29.69]	**<0.001**	7.43 [2.21–25.02]	**0.001**
DSAs at biopsy	4.20 [1.25–14.11]	**0.02**	3.96 [0.84–18.52]	0.080
eGFR at biopsy	0.96 [0.94–0.99]	**0.004**	0.97 [0.94–1.005]	0.094
dd-exoDNA ≥1.9 x 10^−3^	4.61 [1.94–10.95]	**0.001**	3.68 [1.32–10.26]	**0.013**

Bold values indicate statistically significant results (p < 0.05).

## Discussion

Unlike other extensively studied kidney biomarkers [1, 5, 39–41], the role of dd-exoDNA remains largely unexplored, offering an opportunity to deepen our understanding of exosome biology and its clinical implications in kidney transplantation. Studies in heart and lung transplantation suggest that donor-derived exosomes may detect rejection before histological changes, although standardized assays and large-scale validation are still lacking [5, 42, 43]. To our knowledge, this single-center, cross-sectional study is the first to report the detection of donor-specific HLA, particularly donor-derived HLA-DR alleles, in exosomes isolated from KTR serum using dPCR. Although exploratory and proof-of-concept, our findings support the biological plausibility of dd-exoDNA as a promising complementary biomarker of allograft injury.

HLA-DR was selected due to its clinical relevance, as mismatches in this allele play a critical role in kidney allograft rejection and survival [44], and its limited polymorphism allows broad population coverage through validated probes [36, 37].

Moreover, the association between dd-exoDNA levels and biopsy-proven rejection supports its potential complementary role. Patients with TCMR tended to show higher dd-exoDNA levels, a finding that should be interpreted cautiously given the limited subgroup size and wide confidence intervals, particularly in chronic-active forms, which encompass variable degrees of ongoing inflammation and tissue injury. This variability likely reflects biological heterogeneity within TCMR. Increased dd-exoDNA levels may relate to the immunogenic role of renal tubular epithelial cells (RTECs), which can express HLA-DR under pathological conditions, including allograft rejection [45–49]. Previous studies have demonstrated HLA-DR expression in a substantial proportion of tubular cells in TCMR, supporting their role in antigen presentation and immune modulation [47, 49]. As RTECs are a major source of exosomes in the kidney microenvironment, their heightened immunogenic activity may contribute to increased exosomal DNA release in TCMR.

In contrast, the absence of differences between DSA-positive and DSA-negative ABMR cases likely reflects the biological heterogeneity of ABMR, where microvascular injury may occur independently of circulating antibodies. The substantial overlap observed between rejection and non-rejection groups is consistent with the biological heterogeneity of graft injury and supports the interpretation of dd-exoDNA as a complementary biomarker rather than a standalone diagnostic test.

The correlations observed between dd-exoDNA and selected Banff lesions (g, ptc, t) provide additional mechanistic support. These lesions represent endothelial and tubular inflammatory injury, both of which could contribute donor-derived material to the EV compartment. Significantly higher values in tubulitis, glomerulitis, and peritubular capillaritis suggest dd-exoDNA may also derive from endothelial injury, as endothelial cells can upregulate class II MHC expression under inflammatory conditions [50]. These observations align with prior studies of cell-free DNA dynamics [41, 51] and reflect the complexity of exosomal DNA release in both tubular and microvascular compartments. This discrepancy likely reflects differences between continuous lesion scores and categorical diagnoses, as well as the greater biological heterogeneity of ABMR compared with high-grade TCMR. Some biopsies classified as non-rejection despite elevated dd-exoDNA levels showed chronic or injury-related features that did not meet formal Banff rejection criteria, suggesting that dd-exoDNA may capture graft injury-related processes not fully reflected in categorical diagnoses. These observations should therefore be viewed as hypothesis-generating.

In terms of diagnostic performance, dd-exoDNA showed modest discrimination between rejection and non-rejection, consistent with early-phase non-invasive biomarkers [1, 39], particularly in TCMR, where current biomarkers have well-recognized limitations [39, 40, 51]. Predictive values were influenced by the relatively high rejection prevalence in our cohort (47%), whereas sensitivity and specificity indicated moderate diagnostic accuracy. Together, these findings support dd-exoDNA as a complementary biomarker rather than a stand-alone diagnostic tool with performance likely dependent on clinical context and pretest probability. Performance improved in clinically indicated biopsies, where pretest probability of rejection and the severity of histological lesions are typically higher. In contrast, performance in surveillance biopsies was limited, consistent with the small number of rejection cases and the expected challenge of detecting subtle subclinical injury in a cross-sectional design.

Dd-exoDNA positivity (≥1.9 × 10^−3^) remained independently associated with rejection, even after adjustment for potential confounders including biopsy indication, immunosuppressive regimen, DSA status, eGFR at biopsy, and baseline immunological risk (OR 3.68, 95% CI 1.32–10.26, *p* = 0.013). Although statistically significant, the wide confidence intervals indicate limited precision of effect estimates, likely reflecting the modest sample size and the number of rejection events. Accordingly, effect magnitude should be interpreted cautiously and considered exploratory pending validation in larger cohorts. In decision curve analysis, including dd-exoDNA improved net benefit relative to the baseline model across probability thresholds. Clinically relevant benefit was mainly observed at lower thresholds (≈0.20–0.30), which better reflect real biopsy decision ranges, whereas higher thresholds primarily illustrate model behavior.

Beyond DNA quantification, we explored whether EV surface antigen profiles differed between rejection and non-rejection samples. Several immune-related markers (CD3, CD8, CD2, CD40, MCSP, and CD86) showed nominally lower expression in rejection; however, none remained significant after correction for multiple comparisons, underscoring the exploratory nature of the MACSPlex analysis. Despite this, the consistent directionality across markers suggests that EV surface phenotypes may reflect underlying immunological processes during rejection. The trend toward lower CD40 expression in ABMR is biologically plausible, given its role in antigen presentation and B-cell activation, but requires confirmation in larger cohorts. Prior studies have demonstrated that EV surface profiles may carry prognostic information in solid organ transplantation, including in cardiac [7] and kidney transplant [52] settings. Importantly, by leveraging donor–recipient HLA-DR mismatches, our study uniquely assigns graft origin to circulating EV-associated DNA, providing a framework for future integrated immune monitoring.

This work must be interpreted in the context of several limitations. First, the relatively small sample size and cross-sectional design preclude assessment of temporal dynamics and limit subgroup analyses and causal inferences. Second, DNase treatment was not performed prior to vesicle lysis; therefore, the assay quantifies total EV-associated donor-derived DNA rather than intravesicular DNA alone. Although current evidence suggests that a substantial fraction of EV-associated DNA may be localized on the vesicle surface, this approach does not allow definitive discrimination between surface-bound and intraluminal DNA fractions. In addition, the low DNA input limits analytical resolution at low donor fractions, potentially affecting patient-level interpretation and threshold robustness. Third, the reliance on HLA-DRB1 mismatches limits applicability in donor–recipient pairs identical for this locus (10.5% in our cohort), underscoring the need for expanded probe panels targeting additional HLA loci to enable broader clinical implementation. Fourth, the mixed inclusion of surveillance and clinically indicated biopsies generated a non-representative rejection prevalence, which directly influences predictive values. Therefore, PPV and NPV estimates should be considered context-specific and not extrapolated to broader transplant populations without external validation. Moreover, while ROC-based discrimination (AUC) is not mathematically dependent on disease prevalence, the mixed case-mix may introduce spectrum effects that influence AUC estimates; thus, the observed discrimination should also be interpreted as context-specific. Finally, the cost-effectiveness of dd-exoDNA testing remains to be established, given the specialized equipment required. From a practical perspective, assay costs are mainly driven by HLA-specific probes and access to digital PCR technology. Using precipitation-based exosome isolation and dPCR, the workflow can be completed within a single working day, allowing short turnaround times and potential integration into centralized clinical laboratories.

In conclusion, despite these limitations, our findings provide proof of principle that donor-derived DNA can be reliably detected in the EV-enriched fraction of serum and is associated with histological graft injury. As interest in the diagnostic and therapeutic potential of extracellular vesicles continues to grow, dd-exoDNA represents a biologically grounded and technically feasible avenue for non-invasive immune surveillance. Future studies should incorporate longitudinal sampling, mechanistic characterization of exosomal DNA release, integration with established biomarkers like dd-cfDNA, and validation in external cohorts. Such work will determine whether dd-exoDNA can ultimately complement existing tools to refine rejection monitoring and enhance precision in post-transplant care.

## Data Availability

The datasets presented in this study can be found in online repositories. The names of the repository/repositories and accession number(s) can be found in the article/Supplementary Material.
