# Supplementary material for: Discovery of Donor-Derived Exosomal DNA as an Exploratory Biomarker of Kidney Graft Rejection: A Cross-Sectional Study
Source: Transpl Int. 2026 Mar 18;39:16061. doi: 10.3389/ti.2026.16061 (PMC13038620; doi:10.3389/ti.2026.16061)
Supplement: Supplementary file 1 [file DataSheet1.docx]

**This article contains the following supplemental material:**

**-** **Supplemental Figure 1.** ROC curves for rejection diagnosis of eGFR (purple), DSAs (yellow) and dd-exoDNA fraction (green).

**- Supplemental Figure 2.** ROC curves for rejection diagnosis of allograft biopsy.

**- Supplemental Table S1.** MACSPlex analysis of extracellular vesicle surface markers in rejection and non-rejection groups.


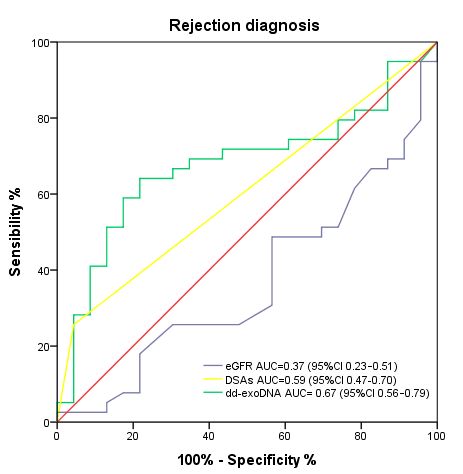


**Supplemental Figure 1. ROC curves for rejection diagnosis of eGFR (purple), DSAs (yellow) and dd-exoDNA fraction (green)** (eGFR; estimated glomerular filtration rate, DSAs; donor-specific antibodies). An area under the curve of 0.37 (95% CI: 0.23–0.51) for distinguishing rejection from non-rejection is seen for eGFR and 0.59 (95% CI: 0.47–0.70) for DSAs, compared to 0.67 (95% CI: 0.56-0.79) for dd-exoDNA.


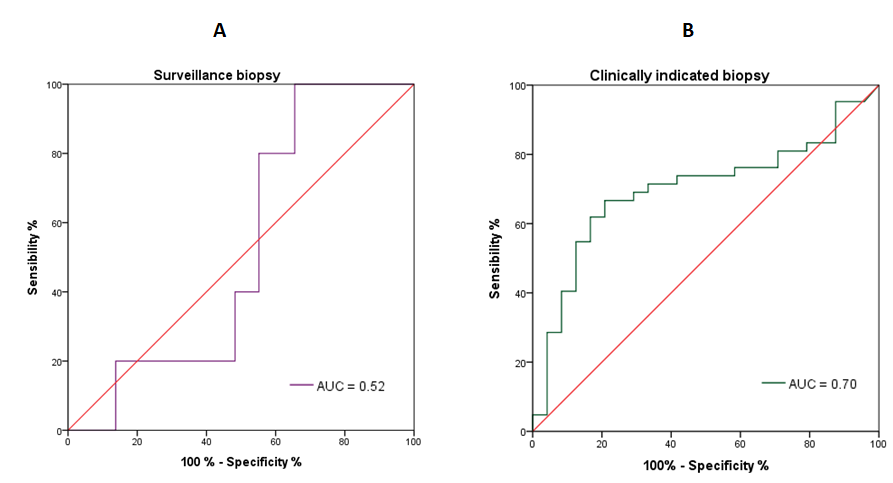


**Supplemental Figure 2. ROC curves for rejection diagnosis of allograft biopsy. (A)** An area under the curve of 0.52 (95% CI: 0.30–0.74) for distinguishing rejection from non-rejection is seen for surveillance biopsy. **(B)** On the contrary, clinically indicated biopsy shows and area under the curve of AUC 0.7 (95% CI: 0.57–0.83).

**Supplemental Table S1.** MACSPlex analysis of extracellular vesicle surface markers in rejection and non-rejection groups.

| **Marker** | **No rejection** | **Rejection** | **p value** | **p value (adjusted)** |  |  |  |  |  |
| --- | --- | --- | --- | --- | --- | --- | --- | --- | --- |
| CD19 | 24.00 (13.75–35.00) | 19.50 (15.00–25.25) | 0.265 | 0.409 |  |  |  |  |  |
| CD4 | 32.25 (21.59–43.66) | 25.78 (17.13–37.19) | 0.187 | 0.368 |  |  |  |  |  |
| CD3 | 26.60 (17.00–34.27) | 20.21 (14.00–28.73) | 0.025 | 0.252 |  |  |  |  |  |
| CD105 | 32.89 (22.54–41.35) | 26.32 (18.07–42.34) | 0.4454 | 0.509 |  |  |  |  |  |
| CD56 | 17.79 (10.59–27.86) | 16.15 (9.69–22.70) | 0.441 | 0.509 |  |  |  |  |  |
| HLA-DR/DP/DQ | 41.47 (30.60–55.52) | 36.04 (25.31–48.80) | 0.125 | 0.368 |  |  |  |  |  |
| CD8 | 52.58 (35.69–69.13) | 41.45 (25.17–55.72) | 0.034 | 0.252 |  |  |  |  |  |
| ROR1 | 30.16 (20.47–39.48) | 23.78 (17.13–33.47) | 0.061 | 0.322 |  |  |  |  |  |
| CD49e | 33.99 (26.32–50.34) | 28.91 (21.30–39.37) | 0.221 | 0.389 |  |  |  |  |  |
| CD25 | 42.21 (24.19–50.82) | 30.49 (20.14–48.08) | 0.184 | 0.368 |  |  |  |  |  |
| CD1c | 34.63 (22.18–47.54) | 26.97 (17.79–43.88) | 0.265 | 0.409 |  |  |  |  |  |
| CD2 | 32.35 (24.72–41.26) | 24.44 (13.24–36.45) | 0.024 | 0.252 |  |  |  |  |  |
| CD40 | 36.15 (26.86–49.25) | 29.56 (20.98–40.02) | 0.026 | 0.252 |  |  |  |  |  |
| CD63 | 138.87 (125.49–160.95) | 139.92 (122.42–151.75) | 0.367 | 0.503 |  |  |  |  |  |
| HLA-ABC | 29.98 (19.58–40.23) | 29.13 (20.02–33.29) | 0.433 | 0.509 |  |  |  |  |  |
| SSEA-4 | 29.15 (22.05–41.89) | 25.87 (17.62–33.39) | 0.140 | 0.368 |  |  |  |  |  |
| CD9 | 97.59 (82.17–109.04) | 98.29 (87.45–111.27) | 0.441 | 0.509 |  |  |  |  |  |
| CD209 | 38.38 (27.80–53.94) | 33.11 (21.42–56.16) | 0.468 | 0.509 |  |  |  |  |  |
| CD41b | 55.95 (43.99–76.70) | 58.17 (42.56–70.35) | 0.895 | 0.895 |  |  |  |  |  |
| CD146 | 26.43 (13.58–37.89) | 21.80 (15.44–28.04) | 0.112 | 0.368 |  |  |  |  |  |
| MCSP | 24.92 (16.60–33.35) | 19.37 (15.62–27.04) | 0.049 | 0.302 |  |  |  |  |  |
| CD81 | 54.34 (46.96–78.85) | 57.47 (46.78–81.18) | 0.780 | 0.802 |  |  |  |  |  |
| CD11c | 27.92 (17.36–41.61) | 21.50 (15.45–33.16) | 0.175 | 0.368 |  |  |  |  |  |
| CD62P | 110.31 (81.88–144.35) | 126.50 (96.16–154.38) | 0.189 | 0.368 |  |  |  |  |  |
| CD42a | 48.57 (37.14–62.51) | 46.33 (36.38–59.23) | 0.391 | 0.509 |  |  |  |  |  |
| CD29 | 66.91 (53.66–79.94) | 64.98 (58.47–81.95) | 0.697 | 0.737 |  |  |  |  |  |
| CD24 | 34.98 (26.35–44.20) | 28.56 (22.28–38.80) | 0.157 | 0.368 |  |  |  |  |  |
| CD86 | 25.95 (16.70–33.92) | 21.19 (12.99–27.21) | 0.028 | 0.252 |  |  |  |  |  |
| CD44 | 40.94 (25.33–54.34) | 29.74 (19.91–44.79) | 0.125 | 0.368 |  |  |  |  |  |
| CD326 | 24.06 (14.74–33.12) | 23.16 (14.85–26.00) | 0.326 | 0.464 |  |  |  |  |  |
| CD133-1 | 25.46 (20.02–36.95) | 23.11 (17.53–28.02) | 0.155 | 0.368 |  |  |  |  |  |
| CD14 | 35.42 (22.09–53.40) | 25.31 (15.59–45.18) | 0.308 | 0.456 |  |  |  |  |  |
| CD20 | 22.38 (14.50–30.76) | 18.78 (11.76–23.28) | 0.131 | 0.368 |  |  |  |  |  |
| CD69 | 32.14 (17.66–43.48) | 22.91 (16.05–39.07) | 0.247 | 0.409 |  |  |  |  |  |
| CD142 | 24.58 (18.97–35.64) | 22.65 (16.77–30.88) | 0.218 | 0.389 |  |  |  |  |  |
| CD45 | 25.25 (14.10–35.99) | 21.99 (14.24–31.33) | 0.407 | 0.509 |  |  |  |  |  |
| CD31 | 26.79 (20.56–37.29) | 23.27 (19.34–30.07) | 0.189 | 0.368 |  |  |  |  |  |

Values are presented as median and interquartile range (IQR). Group comparisons were performed using the Mann–Whitney U test. Unadjusted P-values are shown together with P-values adjusted for multiple comparisons using the Benjamini–Hochberg procedure to control the false discovery rate.
